# Development and Validation of a Gene Expression‐Based Prognostic Model for Patients With Small Cell Lung Cancer Treated With Chemotherapy

**DOI:** 10.1002/mco2.71118

**Published:** 2026-11-01

**Authors:** Tingting Liu, Ting Zhou, Silang Mo, Minghui Ge, Wenjuan Ma, JianHua Zhan, Xueyuan Chen, Yuwen Yang, Yuanzhen Ma, Ningning Luo, Wanglong Deng, Yunjie Song, Ran Ding, Yan Huang, Yunpeng Yang, Wenfeng Fang, Dongsheng Chen, Li Zhang, Yuanyuan Zhao

**Affiliations:** ^1^ Department of Medical Oncology Sun Yat‐sen University Cancer Center Guangzhou China; ^2^ State Key Laboratory of Oncology in South China Guangdong Provincial Clinical Research Center for Cancer Sun Yat‐sen University Cancer Center Guangzhou China; ^3^ State Key Laboratory of Neurology and Oncology Drug Development Jiangsu Simcere Diagnostic Technology Co. Ltd Nanjing China; ^4^ Department of Intensive Care Unit Sun Yat‐sen University Cancer Center Guangzhou China

**Keywords:** gene expression signature, prognostic biomarker, routine clinical specimens, small cell lung cancer, tumor immune microenvironment

## Abstract

Small cell lung cancer (SCLC) remains one of the most aggressive malignancies, with limited tools for predicting outcomes and guiding treatment. We developed an SCLC‐Predictive Risk Score (PRS) model for patients treated with chemotherapy using least absolute shrinkage and selection operator regression. The model comprised five genes and was developed in a training cohort of 50 patients using an innovative 795‐gene panel of tumor‐ and immune‐related genes analyzed on the NanoString nCounter platform. Gene expression was measured in routine clinical formalin‐fixed, paraffin‐embedded SCLC specimens, predominantly puncture specimens (36/50). The SCLC‐PRS model stratified patients into high‐risk (PRS‐H) and low‐risk (PRS‐L) groups; patients in the PRS‐H group had significantly worse overall survival (hazard ratio [HR] = 0.35, 95% confidence interval [CI]: 0.18–0.69; *p* = 0.002) and progression‐free survival (HR = 0.44, 95% CI: 0.23–0.84; *p* = 0.008). The prognostic performance of the model was externally validated in two independent cohorts (GSE60052, *n* = 38; George cohort, *n* = 47). Multivariate Cox analysis confirmed SCLC‐PRS as an independent prognostic factor. PRS‐L tumors exhibit a favorable tumor immune microenvironment, with increased CD45^+^ immune infiltration, reduced Tregs or exhausted CD8^+^ T cells, and upregulated antigen presentation (MHC‐I/II). Overall, the SCLC‐PRS model is an innovative, cross‐platform‐compatible, and clinically applicable prognostic tool with potential utility for risk stratification and personalized treatment.

## Graphical abstract

The SCLC‐PRS model could stratify SCLC patients into high‐risk (PRS‐H) and low‐risk (PRS‐L) groups; PRS‐L patients had significantly longer OS. PRS‐L tumors had increased CD45^+^ immune infiltration, reduced Tregs/exhausted CD8^+^ T cells infiltration, and upregulated antigen presentation (MHC‐I/II) expression.

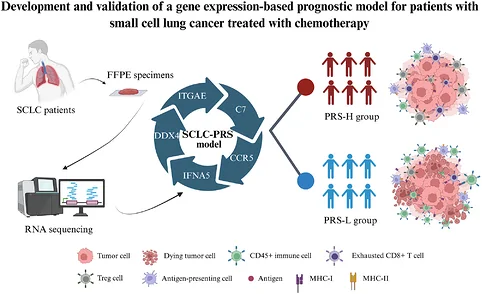

## 1 Introduction

Lung cancer is one of the most common malignancies and a leading cause of cancer‐related mortality worldwide, with small cell lung cancer (SCLC) accounting for approximately 15% of cases [1, 2]. SCLC is characterized by rapid growth, early recurrence, and metastasis [3]. Although immunotherapy has substantially advanced the treatment of SCLC, survival remains poor, and systemic chemotherapy remains a cornerstone of treatment, with chemotherapy plus immunotherapy constituting the standard first‐line therapy [4]. Thus, there is an unmet need for reliable prognostic biomarkers to predict outcomes and improve risk stratification in patients with SCLC.

Given the genomic homogeneity [5] and complex phenotypic plasticity [6] of SCLC, treatment response is difficult to predict based solely on tumor‐intrinsic characteristics. The currently recognized molecular subtypes of SCLC, defined by RNA expression of the lineage‐related transcription factors *ASCL1*, *NEUROD1*, *POU2F3*, and, more controversially, *YAP1* [7, 8], have limited prognostic value [8, 9]. Therefore, a more comprehensive assessment incorporating both tumor‐intrinsic and immune microenvironment may be needed [10].

The tumor immune microenvironment (TIME) influences SCLC development, progression, and treatment response [11, 12]. Transcriptomic characterization of the TIME can provide insights into tumor–immune interactions and facilitate prognostic assessment and identification of biomarkers of treatment response [13]. However, previous gene expression‐based signatures derived from the SCLC TIME have important limitations. First, they have primarily focused on specific gene families involved in T‐cell regulation in the TIME, such as the B7‐CD28 and TNF families [14, 15]. Second, RNA sequencing and model development have relied exclusively on surgical tumor specimens, limiting their applicability to routine clinical samples [14, 15, 16, 17]. In clinical practice, only a small proportion of patients with SCLC are diagnosed early and can undergo surgery (∼4%–12%) [18], whereas approximately 70% of these patients present at an advanced stage. Therefore, developing prognostic models applicable to biopsy specimens is important for extending gene expression‐based risk stratification to a broader population of patients with SCLC.

An innovative 795‐gene NanoString panel covering both tumor‐related and TIME‐related genes, including those involved in immune regulation, immune cell markers, ferroptosis, and pyroptosis, was used to profile gene expression in formalin‐fixed and paraffin‐embedded (FFPE) SCLC specimens obtained in routine clinical practice, including mainly biopsy specimens (78.9%, 75/95). Based on RNA expression data, we developed a five‐gene SCLC‐Predictive Risk Score (PRS) model to stratify the prognosis in patients treated with chemotherapy in a training cohort (*n* = 50). The model was internally validated and subsequently evaluated in two independent external cohorts (GSE60052 cohort, *n* = 38; George cohort, *n* = 47). The SCLC‐PRS is a cross‐platform‐compatible, clinically applicable prognostic model that may facilitate risk stratification across a broad population of patients with SCLC and potentially inform personalized treatment.

## 2 Results

### 2.1 Survival in Patients With SCLC Cannot be Stratified According to Subtype‐A/N/P/Y

In this retrospective clinical study, we included 95 patients with SCLC and collected FFPE tissue samples. The clinical characteristics of the 95 patients and the tissue types of samples are displayed in Table 1. Male patients accounted for 93.7% (*n* = 89), patients with a history of smoking accounted for 89.5% (*n* = 85), and the median age of the cohort was 57 years (range: 36–83). The proportion of puncture specimens was 78.9%. Primary tumor samples accounted for 64.2% of the cases and metastatic samples comprised 35.8%, including 21.1% from proximal lymph node samples and 14.7% from distant metastatic lesions.

**TABLE 1:** Clinical information of 95 SCLC patients.

Variables	Classification	*n* (%)
Age (median, range)		57 (36–83)
Sex	Male	89 (93.7)
	Female	6 (6.3)
Smoking history	Never	10 (10.5)
	Former or ever	85 (89.5)
ECOG‐PS	0–1	87 (91.6)
	2	8 (8.4)
Tumor stage	Limited stage	53 (55.8)
	Extensive stage	42 (44.2)
Chemotherapy	Yes	87 (91.6)
	No	5 (5.3)
	Unknown	3 (3.2)
Chest radiotherapy	Yes	31 (32.6)
	No	61 (64.2)
	Unknown	3 (3.2)
Surgery	Yes	20 (21.1)
	No	72 (75.8)
	Unknown	3 (3.2)
Sampling site	Primary tumor	61 (64.2)
	Proximal lymph node	20 (21.1)
	Distant metastatic lesion	14 (14.7)
Specimens type	Surgical specimens	20 (21.1)
	Puncture specimens	75 (78.9)

Abbreviations: ECOG‐PS, Eastern Cooperative Oncology Group performance status; SCLC, small cell lung cancer.

All FFPE samples were used to detect gene expression at the transcriptional level using the 795‐gene NanoString SCLC‐Immune panel. Following quality control and expression profile quantification, data from 91 samples were entered into subsequent analysis. Based on the RNA expression of the four canonical SCLC subtype markers ASCL1, NEUROD1, POU2F3, and YAP1, unsupervised analysis of 91 SCLC samples assigned patients to different molecular subtypes: subtype‐A (74.7%), subtype‐N (11.0%), subtype‐P (6.6%), and subtype‐Y (7.7%) (Figure 1A). To evaluate the robustness of this clustering approach, we applied the same method to two independent external cohorts, GSE60052 (Figure S1A) and George (Figure S1B) cohorts. The proportions of subtype‐A, N, P, or Y in the George cohort were largely consistent with the previously reported proportions [7].

**FIGURE 1:**
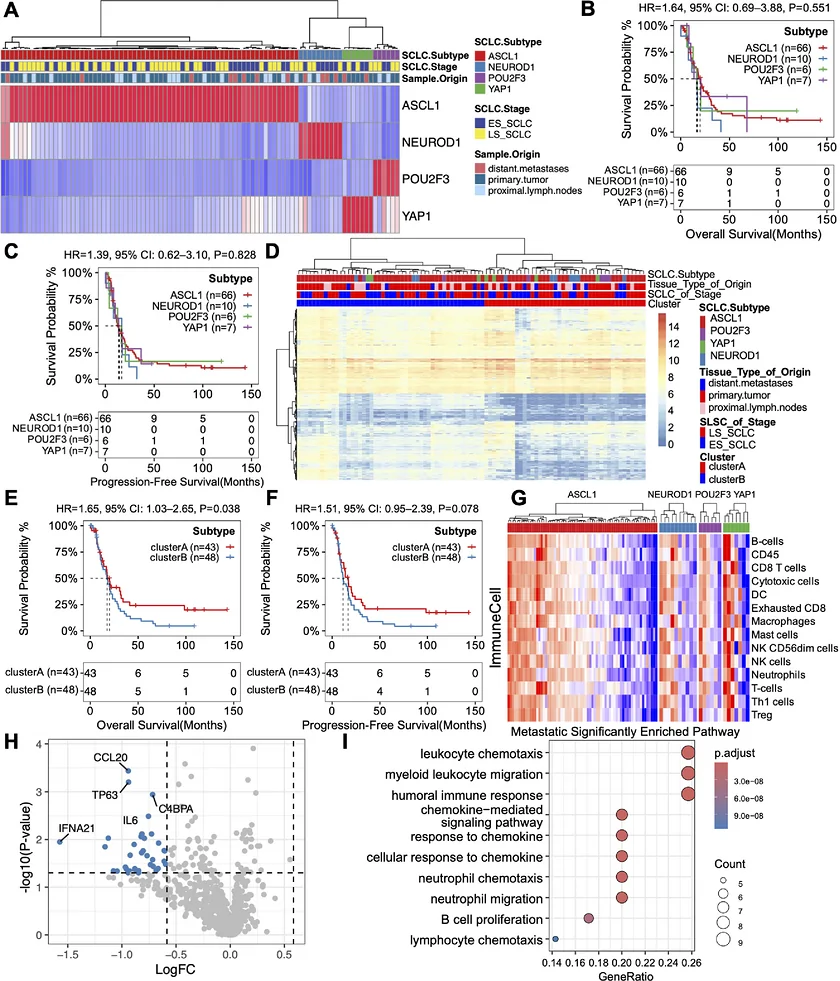
Characteristic and prognosis of gene expression‐based subtypes, and transcriptomic differences between primary and metastatic sites. (A) Unsupervised analysis based on the expression of *ASCL1, NEUROD1, POU2F3*, and *YAP1*. (B and C) Kaplan–Meier curves of OS (B) and PFS (C) among subtypes‐A/N/P/Y. (D) Unsupervised clustering analysis using the consensus clustering method based on gene expression. (E and F) Kaplan–Meier curves of OS (E) and PFS (F) between Cluster A and Cluster B. (G) Unsupervised clustering based on 14 types of immune cell infiltration in subtypes‐A/N/P/Y. (H) Volcano plot displays differentially expressed genes between primary and metastatic sites. (I) Enrichment analysis of differential GO‐BP pathways between primary and metastatic sites. OS, overall survival; PFS, progression free survival; GO‐BP, gene ontology‐biological process; LS, limited stage; ES, extensive stage.

In the SYSUCC cohort, we surveyed the Kaplan–Meier (K‐M) survival curves in patients grouped into four SCLC subtypes (A/N/P/Y). We found that the K‐M survival curve could not stratify patients by overall survival (OS) (hazard ratio [HR] = 1.64, 95% confidence interval [CI]: 0.69–3.88; *p* = 0.551; Figure 1B) or PFS (HR = 1.39, 95% CI: 0.62–3.10; *p* = 0.828; Figure 1C). In the GSE60052 cohort, no significant differences in OS (HR = 0.90, 95% CI: 0.27–2.99; *p* = 0.947) were observed across subtype‐A/N/P/Y (Figure S1C). In the George cohort, compared with subtype‐A/N/P, the subtype‐Y showed poorer prognosis in both OS (HR = 0.97, 95% CI: 0.36–2.63; *p* = 0.005; Figure S1D) and PFS (HR = 0.79, 95% CI: 0.23–2.75; *p* < 0.001; Figure S1E). However, subtype‐Y was present in only two cases, and these results should be interpreted with caution.

### 2.2 Gene Expression Characteristics of Patients With SCLC

First, we conducted an unsupervised clustering analysis based on the expression profiles of 771 genes (excluding 24 housekeeping genes) from the general population using the consensus clustering method. All patients were divided into Clusters A and B (Figure 1D). Prognostic analysis showed that the median OS of Cluster A was significantly longer than that of Cluster B (20.1 vs. 17.5 months, HR = 1.65, 95% CI: 1.03–2.65; *p* = 0.038; Figure 1E). A similar trend was observed for PFS (16.8 vs. 11.5 months, HR = 1.51, 95% CI: 0.95–2.39; *p* = 0.078); however, the difference between these two clusters was not significant (Figure 1F). In Cluster A, significantly upregulated genes were enriched in immune‐related pathways such as natural killer cells, immune response, and T‐cell chemotaxis. The immune killing function was activated, similar to the “hot tumor” state (Figure S1F). In Cluster B, the upregulated genes were significantly enriched in pathways related to JAK‐STAT signal transduction (Figure S1G).

No significant differences were observed in the subtype‐A/N/P/Y distribution between Clusters A and B (Figure 1D). To further understand the TIME characteristics of subtype‐A/N/P/Y in SCLC, we utilized an immune cell scoring algorithm to calculate the infiltration scores for 14 immune cell types [19] and performed unsupervised clustering of these immune cell types in each subtype (Figure 1G). The results revealed that each subtype among subtypes A/N/P/Y contained samples with high and low levels of immune cell infiltration (Figure 1G).

Our results demonstrated significant differences in gene expression between primary tumors and metastatic sites. Compared to primary sites, 36 genes were significantly downregulated in the metastatic sites (Figure 1H). Downregulated genes were mainly enriched in biological pathways related to tumor immunity, such as the chemokine‐mediated signaling pathway, chemokine response, humoral immune response, and B‐cell proliferation (Figure 1I). In addition, the DC and mast cell scores were significantly lower in metastatic lesions (Figure S1H,I).

### 2.3 Development and Validation of the SCLC‐PRS Model

Considering the necessity of chemotherapy as a treatment for SCLC, we developed a chemotherapy‐predictive model to evaluate the survival of patients with SCLC. Given the expression differences between the primary and metastatic lesions in the above analysis, the inclusion criteria for patients used to establish the model were as follows: patients treated with chemotherapy (*n* = 83), those with samples of primary sites (*n* = 57), and those with an OS ≥3 months (*n* = 89). Among the 57 patients with samples from primary sites, six had no chemotherapy record information, and one patient had an OS less than 3 months. Ultimately, 50 patients were included in the SYSUCC training set. The George (*n* = 47) and GSE60052 cohorts (*n* = 38) were used as the validation cohorts. The workflow for the series of tasks, including the construction and validation of the predictive model, is shown in Figure 2A. We adopted a method similar to that developed by Wang et al. [20], and employed the machine learning algorithm the least absolute shrinkage and selection operator (LASSO) and five‐fold cross‐validation to select candidate genes associated with survival in patients undergoing chemotherapy. Based on the HR of candidate genes and their expression levels, we calculated the SCLC‐PRS for each patient. Patients were stratified into two groups using a risk threshold of 1 (median of SCLC‐PRS): PRS‐H (high risk, PRS ≥1) and PRS‐L (low risk, PRS <1). The formula for SCLC‐PRS derived from the model is related to the expression levels of five genes:

$$\begin{array}{c} SCLC-PRS=\left(-1*C7\right)+\left(-1*CCR5\right)+\left(1*DDX4\right)\\ +\left(1*IFNA5\right)+\left(-1*ITGAE\right) \end{array}$$

**FIGURE 2:**
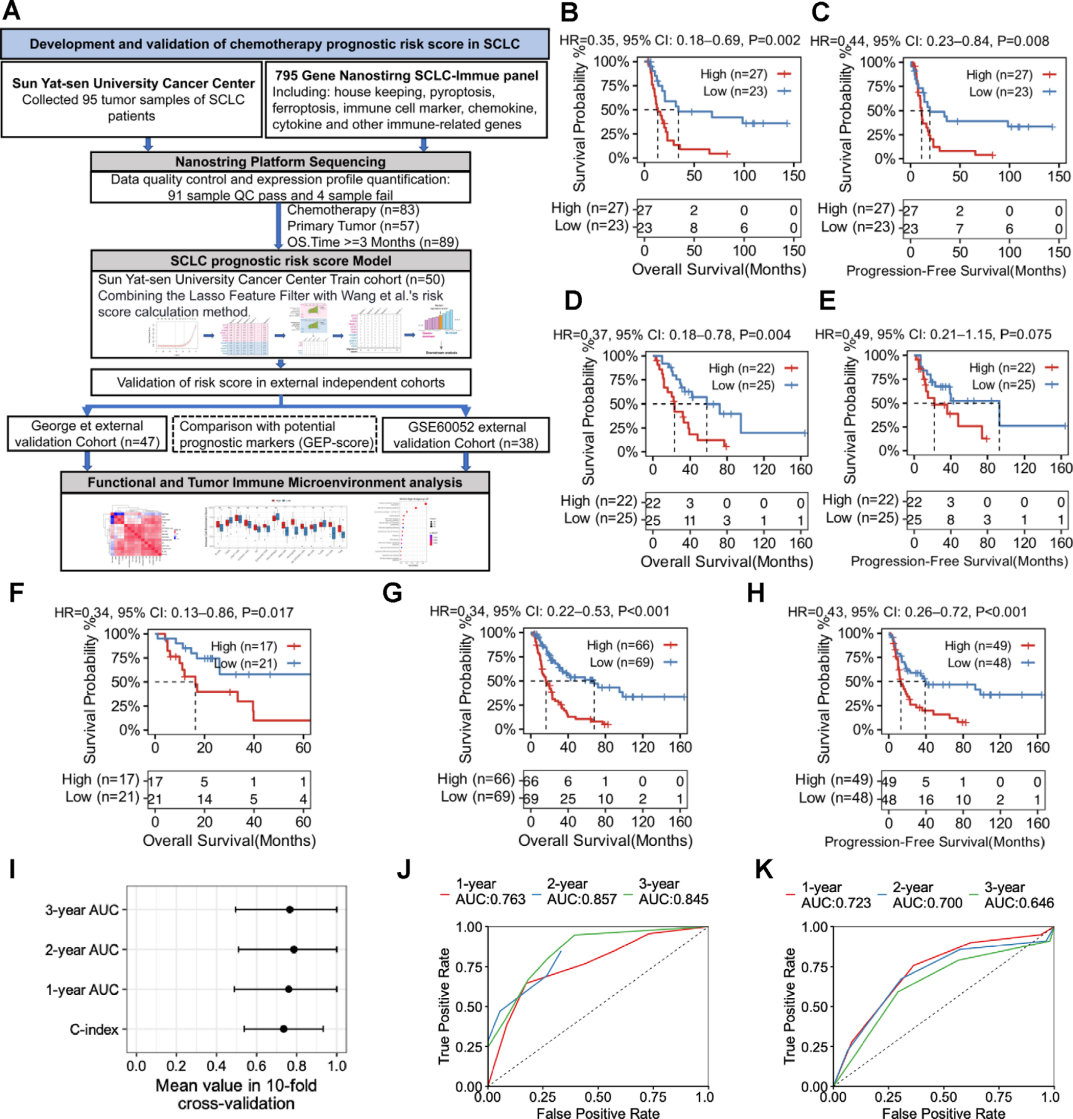
Development and validation of the SCLC‐PRS model. (A) Workflow for the development of the SCLC‐PRS model. (B and C) Kaplan–Meier curves of OS (B) and PFS (C) in PRS‐H and PRS‐L groups in the training cohort (SYSUCC cohort). (D and E) Kaplan–Meier curves of OS (D) and PFS (E) in PRS‐H and PRS‐L groups in the George cohort. (F) Kaplan–Meier curves of OS in the GSE60052 cohort. (G and H) Kaplan–Meier curves of OS (G) and PFS (H) in the combined cohort (SYSUCC, George, and GSE60052 cohorts). (I) Internal 10‐fold cross‐validation analysis of the SCLC‐PRS model in the SYSUCC cohort. (J and K) ROC curve analysis of 1‐, 2‐, and 3‐year survival using the SCLC‐PRS model in the SYSUCC cohort (J) and the combined external cohort (George and GSE60052 cohorts) (K). PRS‐L, predictive risk score‐low; PRS‐H, predictive risk score‐high; ROC, receiver operating characteristic; AUC, area under the curve.

In the SYSUCC cohort, patients in the PRS‐H group had significantly shorter OS (HR = 0.35, 95% CI: 0.18–0.69; *p* = 0.002; Figure 2B) and PFS (HR = 0.44, 95% CI: 0.23–0.84; *p* = 0.008; Figure 2C) than those in the PRS‐L group. In the George cohort, the patients grouped into the PRS‐H group had significantly shorter OS (HR = 0.37, 95% CI: 0.18–0.78; *p* = 0.004; Figure 2D) and showed a trend toward shortened PFS that did not reach statistical significance (HR = 0.49, 95% CI: 0.21–1.15; *p* = 0.075; Figure 2E). In the GSE60052 cohort without PFS, a significant difference also existed in OS between PRS‐H and PRS‐L groups (HR = 0.34, 95% CI: 0.13–0.86; *p* = 0.017; Figure 2F) using the same cutoff. The combined cohort containing all patients who received chemotherapy from the SYSUCC, George, and GSE60052 cohorts showed a significant stratifying in OS (HR = 0.34, 95% CI: 0.22–0.53; *p* < 0.001; Figure 2G) and PFS (HR = 0.43, 95% CI: 0.26–0.72; *p* < 0.001; Figure 2H) between PRS‐H and PRS‐L groups.

To further evaluate the robustness of the SCLC‐PRS model, we performed an internal 10‐fold cross‐validation analysis of the SYSUCC cohort. The internal validation model had a concordance index (*C*‐index) of 0.735, and 1‐, 2‐, and 3‐year AUC values of 0.760, 0.786, and 0.766, respectively (Figure 2I). Receiver operating characteristic (ROC) curve analysis was performed to evaluate the predictive accuracy of the SCLC‐PRS model for OS. In the SYSUCC cohort, the AUC for 1‐, 2‐, and 3‐year OS were 0.763, 0.857, and 0.845, respectively (Figure 2J). In the combined external cohort (George and GSE60052 cohorts), the AUC value for 1‐year OS was 0.723, for 2‐year OS was 0.700, and for 3‐year OS was 0.646 (Figure 2K).

### 2.4 Clinical Assessment of the SCLC‐PRS Model

Multivariate Cox analysis revealed an association of the SCLC‐PRS model and clinical characteristics with survival in the SYSUCC and combined external cohort (George and GSE60052 cohorts). In the SYSUCC cohort, the SCLC‐PRS model was identified as a significantly independent prognostic factor for OS (Figure 3A; HR = 1.35, 95% CI: 1.15–1.62; *p* < 0.001). In the combined external cohort, the SCLC‐PRS model remained an independent prognostic indicator for OS (Figure 3B; HR = 1.24, 95% CI: 1.08–1.43; *p* = 0.003).

**FIGURE 3:**
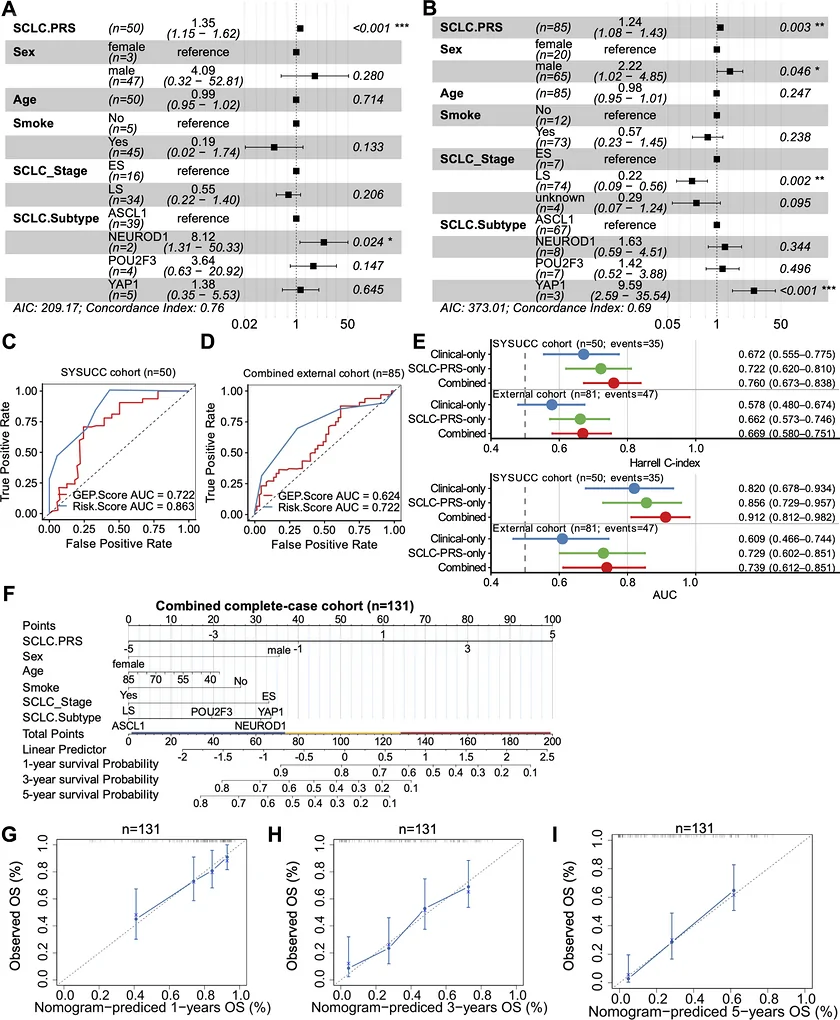
Clinical assessment of the SCLC‐PRS model. (A and B) Multivariable Cox analysis of OS in the SYSUCC cohort (A) and the combined external cohort (George and GSE60052 cohorts) (B). (C and D) ROC curves of the GEP Score and SCLC‐PRS for predicting 2‐year OS in the SYSUCC cohort (C) and the combined external cohort (George and GSE60052 cohorts) (D). (E) Harrell's *C*‐index and IPCW‐estimated AUC value for the clinical‐only model, SCLC‐PRS model, and combined (clinical+PRS) model for predicting 2‐year OS in the SYSUCC cohort and the combined external cohort (George and GSE60052 cohorts). (F) Nomogram for predicting 1‐, 3‐, and 5‐year OS in SCLC in the combined complete‐case cohort (SYSUCC, George, and GSE60052 cohorts). (G, H and I) Calibration plot for predicting 1‐, 3‐, and 5‐year OS in the combined complete‐case cohort. GEP, gene expression profile; IPCW, inverse probability of censoring weighting.

We applied ROC curve analysis to compare the gene expression profile (GEP) Score and SCLC‐PRS model for predicting 2‐year OS. In the SYSUCC cohort, the results demonstrated that the SCLC‐PRS model exhibited a higher AUC than the GEP score (0.863 vs. 0.722; Figure 3C). In the combined external cohort (George and GSE60052 cohorts), the AUC values of the SCLC‐PRS model and GEP score were 0.722 and 0.624, respectively (Figure 3D).

To justify the prognostic effect size of the SCLC‐PRS model, we established a clinical‐only model incorporating conventional clinical parameters, including age, sex, smoking history, SCLC stage, and SCLC subtype. Harrell's *C*‐index and 2‐year inverse probability of censoring weighting (IPCW) estimated AUC for the clinical‐only, SCLC‐PRS, and combined (clinical+PRS) models are summarized in Figure 3E. In the SYSUCC cohort, the clinical‑only model achieved a *C*‐index of 0.672, whereas the SCLC‐PRS model achieved 0.722, and the combined model reached 0.760. The corresponding AUCs for the clinical‐only, SCLC‐PRS, and combined models were 0.820, 0.856, and 0.912, respectively. Paired bootstrap analysis revealed a trend toward improvements in the combined model compared with the clinical‐only model, with a Δ*C*‐index of 0.088 (95% CI: −0.004 to 0.194) and ΔAUC of 0.092 (95% CI: −0.040 to 0.237); neither difference was significant. In the combined external cohort (George and GSE60052 cohorts), the clinical‐only, SCLC‐PRS, and combined model had *C*‐indices of 0.578, 0.662, and 0.669, respectively, with corresponding AUCs of 0.609, 0.729, and 0.739, respectively. The combined model significantly outperformed the clinical‐only model in both *C*‐index (Δ*C*‐index 0.091; 95% CI: 0.012–0.175) and AUC (ΔAUC 0.130; 95% CI: 0.021–0.248) based on paired bootstrap analysis. The differences in the *C*‐index and AUC between the combined and SCLC‐PRS models, or between the SCLC‐PRS and clinical‐only models, were not significant in either the SYSUCC or combined external cohorts.

Next, we integrated the clinical characteristics and SCLC‐PRS to conduct a multivariate analysis, culminating in the creation of a nomogram for predicting 1‐, 3‐, and 5‐year OS in SCLC patients in the combined complete‐case cohort (SYSUCC, George and GSE60052 cohorts) (Figure 3F). The nomogram indicated that SCLC‐PRS had a higher *C*‐index than the other predictors. Moreover, the calibration plots in Figure 3G–I demonstrated good agreement between the predicted and observed survival probabilities for the SCLC‐PRS model in the combined complete‐case cohort at 1, 3, and 5 years.

### 2.5 PRS‐L Group Exhibits Enhanced Expression of Genes Associated With Antitumor Inflammatory Pathways

To investigate the biological mechanisms underlying the predictive effect of the SCLC‐PRS model, we conducted a differential gene expression analysis between the PRS‐H and PRS‐L groups and pathway enrichment studies in 91 patients. We identified 94 genes with significant differential expression between the PRS‐H and PRS‐L groups (Figure 4A). Enrichment analysis of gene ontology‐biological process (GO‐BP) terms revealed that genes significantly upregulated in the PRS‐L group were predominantly enriched in immune‐inflammatory‐related processes, including the immune system process, positive regulation of the immune system process, activation of the immune response, and the B‐cell receptor signaling pathway (Figure 4B). The Kyoto Encyclopedia of Genes and Genomes (KEGG) results mainly reflected tumor‐related pathways such as cell cycle, cell adhesion molecules, and apoptosis (Figure S2A). In the PRS‐H group, significantly upregulated genes were primarily enriched in GO‐BPs associated with JAK‐STAT signaling (Figure 4C) and necroptosis‐related KEGG pathways (Figure S2B).

**FIGURE 4:**
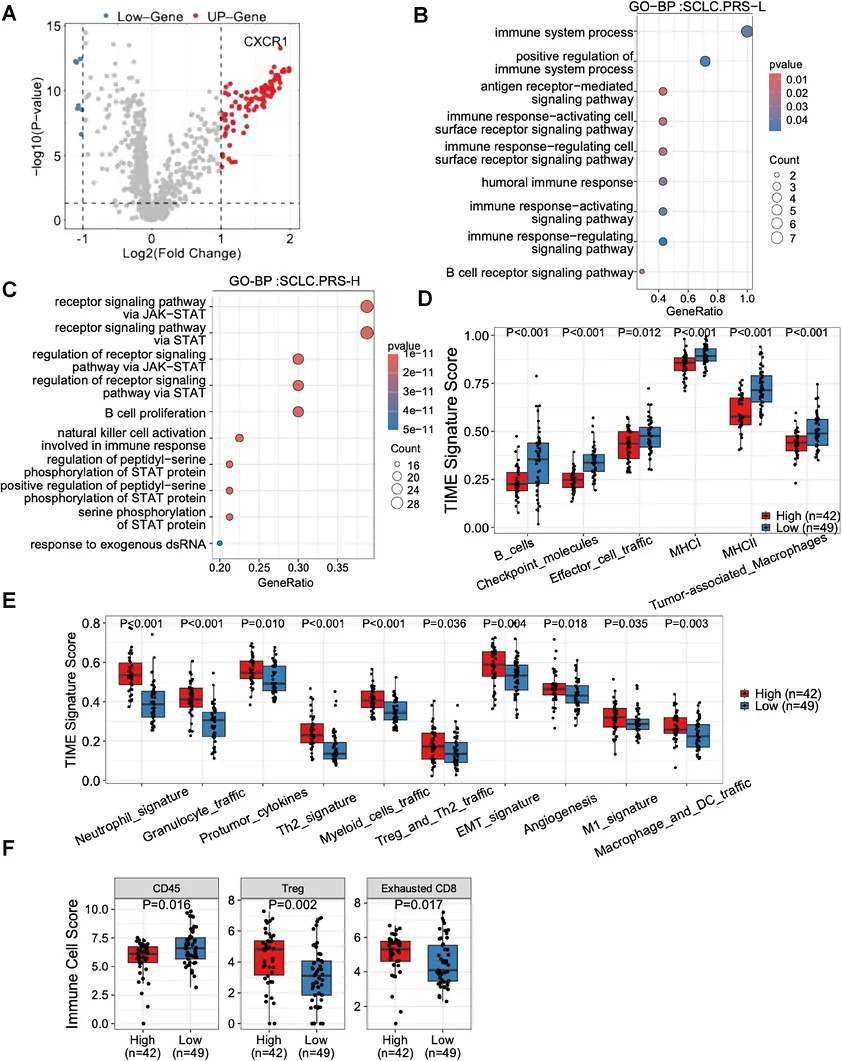
Gene expression profiles and TIME differences between the PRS‐H and PRS‐L groups. (A) Volcano plot displays differentially expressed genes between the PRS‐H and PRS‐L groups. (B and C) GO‐BP enrichment analysis of significantly upregulated genes in the PRS‐L (B) and PRS‐H (C) groups. (D) Comparison of B cells, checkpoint molecules, effector cell traffic, MHC‐I, MHC‐II, and tumor‐associated macrophage scores between the PRS‐L and PRS‐H groups. (E) Comparison of neutrophil signature, granulocyte traffic, pro‐tumor cytokine, Th2 signature, myeloid cell traffic, Treg and Th2 traffic, EMT signature, angiogenesis, M1 signature, macrophage and DC traffic scores between the PRS‐L and PRS‐H groups. (F) Comparison of CD45^+^ cell, Treg cell, and exhausted CD8^+^ T cell scores between the PRS‐L and PRS‐H groups. TIME, tumor immune microenvironment; EMT, epithelial–mesenchymal transition; DC, dendritic cells.

To further characterize TIME in relation to the SCLC‐PRS model, we performed unsupervised clustering based on 28 functional expression features (FGES) [21] in patients, revealing two distinct patterns. In the PRS‐L majority group, significantly upregulated signatures included effector cell traffic, MHC‐I, B cells, and MHC‐II belonging to the antitumor microenvironment cluster (Figure 4D). In contrast, signatures associated with angiogenesis and epithelial–mesenchymal transition (EMT), associated with malignant cell properties, were significantly increased in the PRS‐H majority group (Figure 4E). Furthermore, several pro‐tumor microenvironment signatures, including neutrophil signature, granulocyte traffic, Th2 signature, myeloid cell traffic, macrophage and DC traffic, pro‐tumor cytokines, Treg and Th2 traffic, were markedly elevated in patients with PRS‐H (Figure 4E).

Next, we used a previously validated immune cell scoring algorithm to quantify the infiltration of 14 immune cell types [19]. Cox analysis indicated a significant relationship between CD45^+^ cells, cytotoxic cells, T‐cell scores, and survival (Figure S2C). Further statistical analysis demonstrated that CD45^+^ cell scores were significantly higher in the PRS‐L group (Figure 4F), suggesting a greater degree of lymphocyte infiltration. In the PRS‐H group, the scores for Tregs and exhausted CD8^+^ T cells, which are strongly associated with immune suppression and evasion, were significantly higher (Figure 4F).

Collectively, these findings indicate that PRS‐L tumors are characterized by an immune‐active, inflamed microenvironment, whereas PRS‐H tumors exhibit a more immunosuppressive and “colder” immune landscape accompanied by heightened malignant proliferation and pro‐tumor signaling.

### 2.6 SCLC‐PRS Model as a Potential Predictive Marker for Immunotherapy

Considering that the above results indicate a relationship between the SCLC‐PRS model and immune microenvironment characteristics, we applied the SCLC‐PRS model to predict survival in patients with SCLC receiving immunotherapy. We applied the SCLC‐PRS model to the SCLC immunotherapy cohort (*n* = 17) from Roper's study [22] and stratified them into the PRS‐L and PRS‐H groups based on a constant cutoff value. K‐M survival curves indicated that PRS‐L patients had significantly higher OS than PRS‐H patients (HR = 0.34, 95% CI: 0.14–0.83; *p* = 0.018; Figure 5A). However, it was not effective in PFS (HR = 0.82, 95% CI: 0.29–2.33; *p* = 0.677; Figure 5B). Patients who achieved complete response (CR, *n* = 1) and partial response (PR, *n* = 2) after immunotherapy were in the PRS‐L group (Figure 5C). Finally, we compared the predictive performance of the SCLC‐PRS model, tumor mutation burden (TMB), programmed cell death‐ligand 1 (PD‐L1) expression, and GEP score in evaluating survival after immunotherapy. ROC curve analysis showed that the SCLC‐PRS model had the highest AUC compared with the above immunotherapy prognostic biomarkers (Figure 5D). These findings suggest that patients in the PRS‐L group are more likely to benefit from immune checkpoint blockade therapy. However, owing to the small sample size of the immunotherapy validation cohort, these findings should be considered exploratory.

**FIGURE 5:**
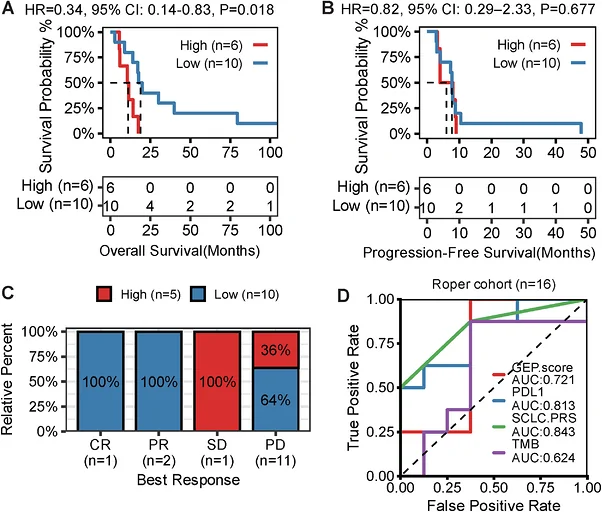
Prognostic value of SCLC‐PRS model in immunotherapy cohort. (A and B) Kaplan–Meier curves of OS (A) and PFS (B) in the PRS‐L and PRS‐H groups. (C) Comparison of best response rate between the PRS‐L and PRS‐H groups. (D) ROC curves of the SCLC‐PRS model, TMB, PD‐L1 expression, and GEP Score for predicting 2‐year OS. TMB, tumor burden; PD‐L1, programmed cell death ligand 1.

## 3 Discussion

In this study, we developed and validated a novel prognostic model (SCLC‐PRS) based on the expression of five immune‐related genes that could independently predict the clinical outcomes of patients with SCLC treated with chemotherapy. The SCLC‐PRS model categorized patients into PRS‐H and PRS‐L groups with distinct PFS and OS, which were significantly longer in the PRS‐L group than in the PRS‐H group. Further analysis revealed that compared to the PRS‐H group, patients in the PRS‐L group exhibited antitumor immune microenvironment characteristics, showing (i) high levels of CD45^+^ cells, low Treg cells, and low levels of exhausted CD8^+^ T‐cell infiltration; (ii) activation of immune response‐related pathway enrichment; and (iii) high expression of antigen presentation‐related molecules MHC‐I and MHC‐II.

In this study, a 795‐gene immune panel on the NanoString nCounter platform was used to profile gene expression in FFPE specimens, with puncture specimens constituting the majority of the total sample set (78.9%). In previous SCLC studies, RNA sequencing data were primarily based on surgically resected specimens [14, 15, 16, 17]. The NanoString nCounter platform successfully detected RNA expression profiles in long‐preserved punctured FFPE specimens, providing technical support for exploring SCLC biomarkers using limited tissue samples.

We observed that patients with subtype‐A, subtype‐N, subtype‐P, or subtype‐Y could be subdivided into the high and low immune cell infiltration groups, which was in agreement with the findings in a recent report published in Cancer Cell: SCLC‐A, SCLC‐N, and SCLC‐I subtypes could be further differentiated into immune “hot” and immune “cold” SCLC [10]. This pronounced intra‐subtype TIME heterogeneity may explain the lack of prognostic differences among the four transcriptional molecular subtypes [8, 9].

The novel SCLC‐PRS model has several advantages: (i) The SCLC‐PRS model could stratify patients into PRS‐H and PRS‐L groups with significantly divergent PFS and OS, suggesting its potential utility in guiding personalized therapeutic decision‐making for patients with SCLC; (ii) the cross‐platform robustness and generalizability of the SCLC‐PRS model were rigorously validated in two multicenter external cohorts (GSE60052 and George cohorts), with different sample types, tumor stage distribution, race, and RNA‐sequencing platforms; (iii) SCLC‐PRS was an independent prognostic factor in both the SYSUCC and combined external cohorts; and (iv) in the combined external cohort, the SCLC‑PRS model provided incremental prognostic value beyond conventional clinical parameters, and its integration with clinical factors yielded further improvements in predictive performance. Therefore, the SCLC‐PRS model has high potential for popularization in clinical applications.

The five‐gene signatures in the SCLC‐PRS model were C7, CCR5, ITGAE, DDX4, and IFNA5. In previous studies, we investigated the roles of these five genes in tumors. C7, a member of the complement system, is essential for the membrane attack complex assembly and is involved in the immune killing process of tumors. This upregulation may suppress tumor growth [23]. CCR5, which is involved in recruiting immune cells in SCLC, is important for tumor antigen recognition and antitumor immune responses [24, 25]. Tissue‐resident memory T cells can usually be identified via the surface marker ITGAE (CD103), which directly kill tumor cells by secreting cytotoxic molecules when activated [26, 27]. DDX4 is a germline factor, and the RNA helicase is conserved in all multicellular organisms. It positively regulates cancer progression, including SCLC, and is closely associated with innate immunity [28]. IFNA5 belongs to the type I interferon (IFN‐I) family. IFN‐I plays a dual role in tumor suppression and progression, and participates in immune regulation and killing [29]. We found that all five genes were immune‐related. Unlike previous studies that focused only on genes involved in T‐cell regulation in TIME, the SCLC‐PRS model exemplifies multiple dimensions of the immune response, including tumor antigen recognition, T‐cell priming and activation, and tumor cell killing. Therefore, these genes may serve as potential therapeutic targets in patients with SCLC.

We also compared the TIME between the PRS‐L and PRS‐H groups in three dimensions: immune cell infiltration, pathway enrichment, and functional gene expression. We found that (i) from the perspective of immune cell infiltration, CD45^+^ cells were more enriched in the PRS‐L group. In contrast, Tregs and exhausted CD8^+^ cells were enriched in the PRS‐H group. Treg cells suppress anticancer immunity by interacting with other immune cells, evading protective immunosurveillance and promoting tumor progression [30, 31]. Exhausted CD8^+^T cells exhibit a loss of killing effect function [32, 33]. The suppressive immune microenvironment in the PRS‐H group might be one of the reasons for the poor prognosis. (ii) From the perspective of pathway enrichment analysis, the PRS‐L group was mainly enriched in the immune system process, positive regulation of the immune system process, activation of the immune response, and the B‐cell receptor signaling pathway. The PRS‐H group showed enrichment mainly in JAK‐STAT and necrosis‐related pathways, indicating that the PRS‐L group had a stronger antitumor immune response than the PRS‐H group. (iii) From the perspective of functional gene expression analysis, the PRS‐L group showed higher expression of MHC‐I and MHC‐II than the PRS‐H group. MHC‐I and MHC‐II belong to the antigen processing and presentation machinery (APM) gene family and facilitate T‐cell recognition and immune‐mediated clearance [34]. Recent studies have also revealed that APM upregulation is strongly correlated with a favorable SCLC prognosis [35, 36, 37, 38]. Furthermore, we observed that the PRS‐L group had a high expression of MHC‐I and MHC‐II, with a favorable prognosis. Taken together, the PRS‐L group exhibited a more active immune microenvironment, whereas the PRS‐H group showed a relatively suppressive immune response. These findings partly explain why the SCLC‐PRS model can predict prognosis.

Our study has some limitations. First, this was a single‐center retrospective study with a limited sample size, which may have reduced the statistical power to detect the incremental prognostic value of the SCLC‐PRS model over conventional clinical variables in the SYSUCC cohort. Second, exploration of the biological mechanisms of C7, ITGAE, and IFNA5 in SCLC is warranted. Third, because of the lack of patients with SCLC treated with immunotherapy in our cohort, we validated the prognostic stratification effect of the SCLC‐PRS model in an external immunotherapy cohort with a limited sample size. The results showed that the model could be used to predict immunotherapy outcomes, warranting further validation. Fourth, the SCLC‐PRS model was developed and validated using primary lung tumor specimens. Therefore, its prognostic value in ES‐SCLC diagnosed using biopsy at the metastatic site remains unclear. Fifth, the performance difference between internal 10‐fold validation and combined external validation may be attributable to the limited external sample sizes and cross‐cohort heterogeneity. Larger prospective multicenter studies are warranted to confirm the incremental prognostic value and clinical applicability of the SCLC‐PRS model, and to improve its generalization.

## 4 Conclusions

SCLC‐PRS model is a cross‐platform‐compatible and clinically practical tool for prognostic assessment that has the potential to transform risk stratification and guide personalized treatment strategies. Future large‐scale prospective studies are warranted to confirm the broad applicability and provide mechanistic insights.

## 5 Materials and Methods

### 5.1 Participants and Study Design

We collected high‐quality FFPE tumor tissues from 95 patients diagnosed with SCLC between April 2004 and July 2021 at the Sun Yat‐sen University Cancer Center (SYSUCC). The date of last follow‐up was February 9, 2023. Among the 95 patients, 75 puncture specimens and 20 surgical specimens were available. Clinical and pathological information such as sex, age, smoking history, tumor location, sampling site, sample type, treatment modalities, tumor stage, and Eastern Cooperative Oncology Group performance status were obtained from electronic medical records. Tumor staging was performed according to the American Joint Committee on Cancer eighth edition staging manual [39] and the Veterans Administration Lung Study Group staging manual [40]. All the clinical data are presented in Table 1. This study followed the Transparent Reporting of a multivariable prediction model for Individual Prognosis or Diagnosis of Artificial Intelligence (TRIPOD+AI) statement [41]. The completed TRIPOD+AI checklist is provided in Supporting Information.

### 5.2 RNA Extraction, Processing, and Quality Checking

Total RNA was extracted from SCLC FFPE specimens using the QIAGEN RNeasy FFPE Kit (QIAGEN GmbH, Hilden, Germany), and was quantified and assessed for quality using a NanoDrop ND1000 Spectrophotometer (Thermo Fisher Scientific) via Qubit quantification and a 4200 quality check. Customized probes were hybridized, and gene expression data were generated using the NanoString nCounter platform. The custom‐designed NanoString panel included 795 genes, with functions broadly categorized into oncogenic signaling pathways, immune regulation, immune cell markers, ferroptosis, pyroptosis, genes reported in the literature related to SCLC prognosis and molecular subtyping, including *ASCL1, NEUROD1, POU2F3*, and *YAP1*; and housekeeping genes.

Data processing and quality control of the expression values were conducted using nSolver 2.6 software following the manufacturer's recommendations [42]. Data were normalized using the geometric mean of endogenous housekeeping genes. The expression levels of 795 genes in the 91 samples that passed quality control are listed in Table S1.

### 5.3 SCLC Classic Subtyping Based on the NanoString nCounter Platform

We classified SCLC into classic subtypes based on the normalized expression of the typing genes ASCL1, NEUROD1, POU2F3, and YAP1 on transcriptomic sequencing platforms (NanoString, RNA‐seq). Using Ward's hierarchical agglomerative clustering method (ward. D2), unsupervised hierarchical clustering of the Euclidean distances for typing gene expression was performed on the respective cohorts. As such, this identified four classic SCLC molecular subtypes characterized by the high expression of signature genes: subtype‐A (ASCL1‐high), subtype‐N (NEUROD1‐high), subtype‐P (POU2F3‐high), and subtype‐Y (YAP1‐high) [7].

### 5.4 Outcomes

The primary outcome of the model development was OS, defined as the time interval between pathological diagnosis and death or the last follow‐up. PFS was a secondary outcome, defined as the time interval between the pathological diagnosis and the first disease progression or death.

### 5.5 Development Cohort and Model Construction

We excluded patients with missing chemotherapy data and enrolled those with primary‑site samples and OS ≥3 months to construct a cross‑platform prognostic model for SCLC patients receiving chemotherapy (*n* = 50). This analysis utilized a gene expression signature calculation method similar to that presented in Wang et al. [20], based on prognostic prediction and gene expression rank ordering principles, and identified 231 of the 795 genes. Subsequently, the LASSO Cox regression algorithm was utilized, alongside five‐fold cross‐validation, to enhance the robustness and generalizability [43]. Five genes with the highest predictive power were identified: C7, CCR5, DDX4, IFNA5, and ITGAE. For the selected prognostic marker genes, prognosis hazard scores were assigned based on their HR from single‐factor Cox survival analysis (1 for HR >1, −1 for HR ≤1), similar to that previously described [44]. Each gene was assigned an expression level score based on its relative expression in the cohort (1 for > median, −1 for ≤median). The risk coefficient for each candidate gene in each sample was calculated using the following formula: *I* (*g*) = 1 if HR of gene “*g*” >1; otherwise, *I*(*g*) = −1. *E* (*g*, *j*) = 1 if *V* (*g*, *j*) > median (*Vg*), and *E* (*g*, *j*) = −1 if *V* (*g*, *j*) ≤median (*Vg*). *L, g, j*, and *V* represent the label, gene, sample, and gene expression, respectively:

$$For\;Gene\;g,L_{g,j}=I_{\left(g\right)}\times E_{\left(g,j\right)}$$

Subsequently, the cumulative sum of individual gene risk coefficients was computed for each sample to derive a composite risk score. Patients were divided into high‐risk and low‐risk groups based on the distribution of risk scores (≥1 or <1), effectively differentiating between high‐risk and low‐risk patient populations.

### 5.6 External Independent Validation Cohorts

We collected and processed transcriptomic data and clinical information from two multicenter SCLC patient cohorts: the GSE60052 cohort [45] and George cohort [5]. The GSE60052 cohort comprised 79 archived surgical FFPE specimens, with participants including both Chinese and Caucasian patients, predominantly at Stage III (56%) and Stage IV (4%). The George cohort included 81 freshly frozen surgical specimens, with participants who were mostly White and predominantly at Stage I (42%) and Stage II (20%). Transcriptomic data from both cohorts were obtained using next‐generation sequencing (NGS) platforms. The acquired fragments per kilobase million sequencing data were converted to transcript per million (TPM) format and further transformed using Log2(TPM+1) for subsequent analysis. After applying the prespecified eligibility criteria for model development, 38 patients from the GSE60052 cohort and 47 patients from the George cohort were included in the external evaluation. To evaluate the generalizability of the SCLC‐PRS model, the prespecified SCLC‐PRS formula and risk threshold were applied without refitting in all external cohorts.

### 5.7 Model Performance Evaluation

Discriminative ability was assessed using Harrell's *C*‐index and time‐dependent ROC curves with corresponding time‐dependent areas under the ROC curves (AUCs). For internal validation, the predictive performance of the SCLC‐PRS model was assessed via 10‐fold cross‐validation using Harrell's *C*‐index and time‐dependent ROC curves. For external validation, the predictive performance of the SCLC‐PRS model was assessed using time‐dependent ROC curves and AUC. Time‐dependent ROC curves and AUC were used to evaluate the SCLC‐PRS model and other prognostic markers, including the T‐cell inflamed GEP score [46], TMB, and PD‐L1 expression. To assess the incremental prognostic value of SCLC‐PRS beyond conventional clinical variables, Harrell's *C*‐index and the 2‐year time‐dependent AUC estimated using the IPCW were calculated for the clinical‐only, SCLC‐PRS, and combined (clinical+PRS) models. Pairwise differences in these metrics were quantified using paired nonparametric bootstrap analysis (5000 replicates). The differences were reported together with the 95% bootstrap confidence intervals, defined by the 2.5th and 97.5th percentiles of the bootstrap distributions. A difference was considered statistically significant when its 95% confidence interval excluded zero. Calibration of the SCLC‐PRS model was assessed using calibration plots, which graphically depicted the agreement between the predicted and observed survival probabilities at 1‑, 3‑, and 5‑year time points.

### 5.8 Differential Expression Analysis and Gene Set Functional Enrichment Analysis

Differential expression analysis considered fold changes and *p*‐values from the Wilcoxon test, defining genes with fold changes greater than 2 and *p*‐values less than 0.05, were considered significantly differentially expressed. Based on the filtered upregulated or downregulated genes, gene pathway enrichment analysis was conducted using the clusterProfiler R package (version 4.0) [47], including GO enrichment [48] and KEGG pathway analysis [49].

### 5.9 Tumor Immune Microenvironment Analysis

We used Danaher's method to assess immune cell infiltration, evaluating the infiltration of 14 types of immune cells in tumor tissues through the expression of 60 marker genes [19]. Cell type scores were calculated using a simple average of the logarithmic transformation of the normalized expression levels of marker genes.

We obtained a list of gene expression for the 28 functional expression features (FGES) representing the cellular and functional characteristics of the reactive TIME, as proposed by Bagaev et al.’s study [21]. The scores for each feature were calculated using the “ssGSEA” package in R to assess each characteristic. Twenty‐eight FGES expression patterns encompass most components associated with the TIME and malignancy‐related characteristics that either promote or inhibit tumor progression. These signatures were categorized into four clusters: antitumor microenvironment, pro‐tumor microenvironment, angiogenesis fibrosis, and malignant cell properties.

### 5.10 Public SCLC Cohort for Immunotherapy

To extend the testing of the predictive accuracy of the SCLC‐PRS model for immunotherapy, we accessed the transcriptomic sequencing and clinical data of 17 patients with SCLC who received the PD‐L1 antibody, durvalumab, in combination with olaparib from Roper's study [22] (the Roper cohort).

### 5.11 Biostatistics

All statistical analyses and graphical visualizations were conducted using R software (version 4.1.3) and its associated packages. Categorical distributions were compared using the chi‐square test, unless otherwise specified. Continuous variables were compared using the Student's *t*‐test or Wilcoxon rank‐sum test. The consensus clustering matrix and cumulative distribution function (CDF) curve analysis were used to determine the optimal number of clusters for the unsupervised clustering analysis. The K‐M method was used to obtain PFS and OS curves with the log‐rank test. Univariate and multivariate Cox proportional hazards regression analyses were carried out with the “survival” package (version 3.4‐0) to obtain the HRs and 95% CIs of risk indicators. Statistical significance was defined as a two‐sided *p*‐value of less than 0.05.

## Author Contributions

Conception and design: Tingting Liu, Ting Zhou, Silang Mo, Minghui Ge, Dongsheng Chen, Li Zhang, Yuanyuan Zhao. Data acquisition: Tingting Liu, Ting Zhou, Silang Mo, Wenjuan Ma, Jianhua Zhan, Xueyuan Chen, Yuwen Yang, Yuanzhen Ma, Yan Huang, Yunpeng Yang, Wenfeng Fang, Li Zhang, Yuanyuan Zhao. Analysis and interpretation of data: Tingting Liu, Ting Zhou, Silang Mo, Minghui Ge, Ningning Luo, Wanglong Deng, Yunjie Song, Ran Ding. Writing, review, and revision of the manuscript: All authors. All authors have read and approved the final manuscript.

## Funding

This study was supported by the Noncommunicable Chronic Diseases‐National Science and Technology Major Project (Project No. 2024ZD0519700, Subject No. 2024ZD0519705).

## Ethics Statement

The study was conducted in accordance with the Declaration of Helsinki (as revised in 2013). The study protocol was approved by the Institutional Review Board of SYSUCC (ID: B2021‐030‐01), and written informed consent was obtained from each participant.

## Conflicts of Interest

Authors Minghui Ge, Ningning Luo, Wanglong Deng, Yunjie Song, Ran Ding, and Dongsheng Chen are the employees in Jiangsu Simcere Diagnostic Technology Co. Ltd. The remaining authors declare no conflicts of interest.

## Supporting information




**Supporting file 1**: mco271118‐sup‐0001‐TableS1.xlsx

**Supporting file 2**: mco271118‐sup‐0002‐SuppMat.docx

## Data Availability

The NanoString transcriptomic data reported in this paper have been deposited in the OMIX database at the China National Center for Bioinformation ‐ National Genomics Data Center (CNCB‐NGDC) (https://ngdc.cncb.ac.cn/omix/preview/pJlwSDxJ, ID: OMIX017871).
