# Supplementary material for: Development and Validation of a Gene Expression‐Based Prognostic Model for Patients With Small Cell Lung Cancer Treated With Chemotherapy
Source: MedComm (2020). 2026 Nov 1;7(11):e71118. doi: 10.1002/mco2.71118 (PMC13725213; doi:10.1002/mco2.71118)
Supplement: Supplementary file 1 [file MCO2-7-e71118-s002.docx]

**Title:** Development and validation of a gene expression-based prognostic model for patients with small cell lung cancer treated with chemotherapy

**Authors:** Tingting Liu ^1,2#^, Ting Zhou ^1,2#^, Silang Mo ^1,2#^, Minghui Ge ^3#^, Wenjuan Ma ^2,4^, JianHua Zhan ^2^, Xueyuan Chen ^1,2^, Yuwen Yang ^1,2^, Yuanzhen Ma ^1,2^, Ningning Luo ^3^, Wanglong Deng ^3^, Yunjie Song ^3^, Ran Ding ^3^, Yan Huang ^1,2^, Yunpeng Yang ^1,2^, Wenfeng Fang ^1,2^, Dongsheng Chen ^3*^, Li Zhang ^1,2*^, Yuanyuan Zhao ^1,2*^

**Affiliations:**

^1^ Department of Medical Oncology, Sun Yat-sen University Cancer Center, Guangzhou, China

^2^ State Key Laboratory of Oncology in South China, Guangdong Provincial Clinical Research Center for Cancer, Sun Yat-sen University Cancer Center, Guangzhou, China

^3^ State Key Laboratory of Neurology and Oncology Drug Development, Jiangsu Simcere Diagnostic Technology Co., Ltd., Nanjing, China

^4^ Department of Intensive Care Unit, Sun Yat-sen University Cancer Center, Guangzhou, China

^#^Tingting Liu, Ting Zhou, Silang Mo and Minghui Ge contributed equally to this work

***Corresponding to:**

Yuanyuan Zhao, MD, Department of Medical Oncology, Sun Yat-sen University Cancer Center, 651 Dongfeng East Road, Guangzhou, Guangdong 510060, China. E-mail: [zhaoyy@sysucc.org.cn](mailto:zhaoyy@sysucc.org.cn)

Li Zhang, MD, Department of Medical Oncology, Sun Yat-sen University Cancer Center, 651 Dongfeng East Road, Guangzhou, Guangdong 510060, China. E-mail: [zhangli@sysucc.org.cn](mailto:zhangli@sysucc.org.cn)

Dongsheng Chen, MD, State Key Laboratory of Neurology and Oncology Drug Development, Jiangsu Simcere Diagnostic Technology Co., Ltd.,Nanjing, China. E-mail: dongsheng.chen@simceredx.com

**Supplementary Figures and tables**

**Figure S1. Characterization and prognosis of SCLC subtypes in the GSE60052 and George cohort, functional pathway enrichment between the cluster A and cluster B, and immune cell alterations between primary and metastatic sites.**

**Figure S2. KEGG pathway enrichment in PRS‑H and PRS-L groups, and immune cell‑related Cox regression analysis.**

**Table S1. The custom-designed 795 genes Nanostring Panel**

Figure S1. Characterization and prognosis of SCLC subtypes in the GSE60052 and George cohort, functional pathway enrichment between the cluster A and cluster B, and immune cell alterations between primary and metastatic sites. (A and B) Unsupervised analysis based on the expression of *ASCL1, NEUROD1, POU2F3* and *YAP1* in the GSE60052 cohort (A) and the George cohort (B). (C) Kaplan-Meier curves of OS among subtypes-A/N/P/Y in the GSE60052 cohort. (D and E) Kaplan-Meier survival curves of OS (D) and PFS (E) among subtype-A/N/P/Y in the George cohort. (F and G) GO-BP pathway enrichment analysis of significantly upregulated genes in the cluster A (F) and cluster B (G). (H and I) Differences of DC score (H) and mast cell score (I) between SCLC primary and metastatic sites. DC, dendritic cell. OS, overall survival; PFS, progression free survival; HR, hazard ratio; CI, confidence intervals.

Figure S2. KEGG pathway enrichment in PRS‑H and PRS-L groups, and immune cell‑related Cox regression analysis. (A and B) KEGG pathway enrichment analysis of significant upregulated genes in the PRS-L (A) and PRS-H (B) group. (C) The Cox univariate analysis for 14 immune cell types. KEGG, Kyoto Encyclopedia of Genes and Genomes.
